# An In Vitro System Mimics the Intestinal Microbiota of Striped Beakfish (*Oplegnathus fasciatus*) and Inhibits *Vibrio alginolyticus* by *Limosilactobacillus reuteri*-Derived Extracellular Vesicles

**DOI:** 10.3390/ani14121792

**Published:** 2024-06-14

**Authors:** Bao-Hong Lee, Yeh-Fang Hu, Sofia Priyadarsani Das, Yu-Ting Chu, Wei-Hsuan Hsu, Fan-Hua Nan

**Affiliations:** 1 Department of Aquaculture, National Taiwan Ocean University, Keelung 202301, Taiwan; f96b47117@ntu.edu.tw (B.-H.L.); yehfanghu@mail.ntou.edu.tw (Y.-F.H.); das.sofia@gmail.com (S.P.D.); ytchu@mail.ntou.edu.tw (Y.-T.C.); 2 Department of Food Safety/Hygiene and Risk Management, National Cheng Kung University, Tainan 701401, Taiwan

**Keywords:** extracellular vesicles, *Limosilactobacillus reuteri*, microbiota, *Oplegnathus fasciatus*, *Vibrio* spp.

## Abstract

**Simple Summary:**

Lactic acid bacteria (LAB) are often used to improve the probiotic content in aquaculture organisms. Although LAB were fed to aquaculture organisms, these LAB were often not detected when analyzing the gut microbiota. While the intestinal environment was indeed improved, this indicates that the effect of LAB on aquaculture organisms may not occur through the bacteria themselves, but rather through substances such as extracellular vesicles involved in these processes. This study will utilize an in vitro cultured intestinal bacteria system to exclude host interference and investigate the potential of *L. reuteri*-derived EVs for regulating the intestinal microbiota.

**Abstract:**

Extracellular vesicles (EVs) are functional substances secreted by microbes and host cells, and it has been discovered that they participate in the interactions between different microorganisms. Our recent findings indicate that *Limosilactobacillus reuteri*-derived EVs have the potential to improve the intestinal microbiota of *Oplegnathus fasciatus* fish and inhibit pathogenic bacteria. Previous research has reported that the host intestinal cells play a regulatory role in the intestinal microbiota. This suggested that to investigate the mechanisms through which *L. reuteri*-derived EVs regulate the intestinal microbiota, a system that excludes interference from host intestinal cells should be established. In this study, an in vitro cultured intestinal bacteria system, without host factors, was used to simulate the intestinal microbiota of *O. fasciatus* fish. After adding *L. reuteri*-derived EVs to the system, the changes in the microbiota were analyzed. The results showed that *L. reuteri*-derived EVs effectively reduced the abundance of *Vibrio* spp. In the results of the in vitro experiments, it was also observed that *L. reuteri*-derived EVs have the ability to inhibit *Vibrio alginolyticus*. We further sequenced the small RNA contained in *L. reuteri*-derived EVs and found that these small RNAs can interfere with genes (*LysR, pirin, MIpA/OmpV, CatB,* and *aspartate-semialdehyde dehydrogenase*) related to the growth of *V. alginolyticus*. Taken together, the results indicate that in the absence of host involvement, the small RNAs present in *L. reuteri*-derived EVs have the function of inhibiting pathogenic bacteria and exhibit the potential to regulate the intestinal microbiota.

## 1. Introduction

There are many types of bacteria in the animal intestinal tract. If these bacteria can successfully adhere to the surface of intestinal mucosal cells and subsequently proliferate in the intestine, they are referred to as intestinal bacteria. Intestinal bacteria can be roughly categorized into three major types, including the (i) normal flora that resides in the host’s intestinal tract, (ii) beneficial probiotics that contribute to the host’s health, and (iii) pathogenic bacteria that opportunistically infect the host. Through interactions among themselves and with the host, these bacteria coexist in the host’s intestinal tract, forming a stable and complex intestinal microbiota [1]. The complex interactions between probiotics and pathogenic bacteria play a crucial role in stabilizing the intestinal microbiota in the host and preventing the overgrowth of pathogenic bacteria. These interactions include competitive exclusion, the barrier effect, bacterial interference, and antagonism [2].

In recent years, numerous studies have confirmed that a healthy intestinal microbiota can maintain the physiological state of aquaculture organisms and alleviate the impact of harsh environments (such as hydrogen sulfide, ammonia, and nutrients) that may lead to microbiota change and immunity alleviation [3]. When the gut microbiome is imbalanced, aquaculture organisms are more susceptible to infections from pathogens and parasites. For instance, in high-density cage systems, a significant change in the intestinal microbiota of farmed Cobia fish (*Rachycentron canadum*) has been observed, namely, an increase in the prevalence of pathogenic bacteria (such as *Vibrio* spp. and *Photobacterium* spp.) that often leads to diseases in farmed Cobia fish [4]. In the same study, both pathogenic *Pelomonas* and *Fusobacterium* spp. in the intestinal tract of milkfish were increased [4]. Changes in the aquaculture environment can also alter the composition and distribution of the microbiota in salmon, specifically *Firmicutes*, *Proteobacteria*, *Bacteroidetes*, and *Tenericutes* [5].

The results mentioned above indicate that intestinal health significantly influences the growth status of aquaculture organisms. Therefore, exploring effective methods to maintain intestinal health is an essential research strategy. Intestinal cell activity plays an extremely important role in the growth of aquaculture organisms. Research has shown that the enzymes secreted by the intestines exhibiting a healthy microbiota can enhance the metabolic response to feed [6]. *Lactobacillus casei* has been found to enhance the immune activity of zebrafish, providing protective effects against *Aeromonas veronii* infection [7]. Different strains of lactic acid bacteria (LAB) were administered to zebrafish through immersion, and the adhesion ability and infection prevention capabilities of these strains in the zebrafish intestine were compared. The results revealed 49 strains of *Lactobacillus,* categorized into three major types, based on their distribution in the intestine: mucus type, mucosa type, and hybrid type. Among these LAB, the *Lactobacillus brevis* (hybrid type) demonstrated the highest resistance to *Aeromonas hydrophila* infection in zebrafish [8].

It is challenging to maintain normal gut microbiota solely by supplementation with probiotics. External probiotics often struggle to survive gastric acid and establish substantial colonization in the intestines. The composition of gut microbiota is also influenced by factors such as living conditions, dietary habits, health status, and the availability of probiotic nutritional sources like polysaccharides and oligosaccharides. Generally, LAB should be considered protective for aquaculture hosts under several conditions, including (i) administration to aquaculture organisms in a live state, (ii) the orally administered live LAB must safely reach the intestines and maintain activity, and (iii) these live LAB must be able to colonize and grow in the intestines.

Although commercially available LAB powders possess high live bacterial counts, the cost remains a significant consideration for aquaculture farmers, making it difficult to implement these powders in aquaculture. Therefore, many farmers choose to ferment feed to produce live LAB for feeding. However, this method may lead to significant contamination during the fermentation process, especially when farmers lack adequate equipment. Our recent research has found that LAB has the potential to improve the intestinal microbiota and suppress pathogens in farmed fish (*O. fasciatus*) [9].

Sha et al. (2016) found that the supplementation of different probiotics (*Lactobacillus pentosus, Enterococcus faecium*), along with a supernatant containing a high concentration of LAB-derived EVs after removal of the bacterial cells, significantly increased the abundance of *Actinobacteria* in the intestine of shrimp [10]. The fermentation broth containing abundant EVs (which are considered as waste from food fermentation factories) could be developed. It could facilitate the bridging of connections between aquaculture farmers and food fermentation factories for application in the prevention and control of diseases in aquaculture organisms.

*Limosilactobacillus reuteri*-derived EVs have been found to upregulate proportions of intestinal *Bifidobacterium, Blautia,* and *Dorea* species and downregulate pathogenic bacterial growth (*Acinetobacter, Escherichia_Shigella,* and *Megasphaera*) in *O. fasciatus*. While it is known that the antibacterial ability is lost when the small RNA is removed from *L. reuteri*-derived EVs, further research is still required to fully understand the detailed interactions of *L. reuteri*-derived EVs with intestinal microorganisms [9]. This study utilized an in vitro cultured intestinal bacteria system to exclude host interference and investigate the potential for *L. reuteri*-derived EVs in regulating the intestinal microbiota.

## 2. Materials and Methods

### 2.1. Materials

Gifu anaerobic broth (GAM) was obtained from Himedia Laboratories (Mumbai, India). Brain Heart Infusion (BHI) medium was purchased from Thermo Fisher Scientific (Waltham, MA, USA). Acetic acid, propionic acid, isobutyric acid, and butyric acid were purchased from Sigma (Sigma-Aldrich, St. Louis, MO, USA). The QIAamp PowerFecal DNA kit was purchased from Qiagen (Hilden, Germany).

### 2.2. Samples Preparation

*Limosilactobacillus reuteri* was isolated from lamb feces; the *L. reuteri*-fermented diet enriched with EVs was found to inhibit pathogens and accelerate fish growth in our previous study [9]. This LAB was inoculated into de Man, Rogosaand Sharpe (MRS) broth (BD Biosciences, San Jose, CA, USA), or MRS agar at 37 °C. Briefly, the *L. reuteri* was sub-cultured for activation in MRS broth, and this activated bacteria was re-inoculated (1%) into fresh MRS broth (400 mL) for cultivation under anaerobic conditions at 37 °C for 24 h. The bacteria in the MRS medium were removed by centrifugation at 3000× *g* for 15 min. Bacterial debris and intact organelles in the culture medium were further removed by centrifugation at 35,000× *g* for 60 min. The supernatant was then subjected to ultracentrifugation at 200,000× *g* for 60 min, according to the methods of our previous study [11,12]. The pellets were resuspended in PBS. The particle number of extracellular vesicles (EVs) and their size distribution were detected by a nanoparticle tracking analyzer (Nano-ZS 90 dynamic light scattering, Malvern Panalytical, Malvern, UK).

### 2.3. Culture of Intestinal Bacteria and Microbiota Sequencing

Culture of intestinal bacteria: The in vitro intestinal microbial community utilized in the experiments was cultured and maintained in a simulator of an intestinal microbial ecosystem, as previously described [13,14]. Intestinal samples were collected from the colons of 12 *Oplegnathus fasciatus* (381 ± 14.7 g). The fresh stool samples (5.8 g) were homogenized with 18 mL of GAM broth. The fecal solution was inoculated into the fresh culture broth and incubated at 30 °C for 24 h under anaerobic conditions. Additionally, a feed solution was added three times daily to provide digested nutrition for the colon microorganisms. After adaptation for 3 weeks, stable microbial communities were obtained. The *L. reuteri*-derived EVs samples were added into an in vitro cultured intestinal bacteria system for 48 h, and the microbial community was sequenced [14,15].

Microbiota sequencing: The culture medium was collected and immediately soaked in liquid nitrogen and stored at −80 °C for subsequent use. The total genomic DNA was extracted from the samples using a QIAamp PowerFecal DNA kit (Hilden, Germany) for full-length 16S rRNA amplicon sequencing. PCR amplification was performed using the 16S primer F (5′-AGRGTTYGATYMTGGCTCAG-3′) and R (5′-RGYTACCTTGTTACGACTT-3′) and purified using AMPure PB Bead (PacBio, CA, USA). The SMRTbell adapter was attached to the purified PCR product and sequenced full-length 16S rRNA using the PacBio RS II SMRT DNA sequencing system (Pacific Biosciences, Menlo Park, CA, USA), employing the P6-C4 chemistry. Repeat sequences were organized to generate circular consensus sequencing (CCS). CCS data was analyzed using DADA2 to produce amplicon sequence variants (ASVs). Species information was obtained by comparing the data with the NCBI database [14,15].

### 2.4. The Effects of L. reuteri-Derived EVs against Vibrio Alginolyticus

*Vibrio alginolyticus* (BCRC12829) was purchased from Bioresource Collection and Research Center (BCRC) in Taiwan (Hsinchu, Taiwan) and cultured in a medium (formula: NaCl 30 g, soytone 5 g, and tryptone 15 g in 1 L distilled water), with or without EVs treatment, in an aerobic environment at 37 °C. Three independent samples were analyzed for each experiment, and the OD600 was measured after culturing the cells for different time periods [9].

### 2.5. Prediction of Small RNA Obtained from L. reuteri-Derived EVs

Sample libraries were prepared using the QIAseq miRNA Library Kit (QIAGEN), according to the manufacturer’s protocols. Adaptors were ligated sequentially to the 3′ and 5′ ends of miRNAs. Subsequently, universal cDNA synthesis with UMI assignment, cDNA cleanup, library amplification, and library cleanup were performed. Libraries were sequenced on an Illumina instrument (75-cycle single-end read, 75SE). Initially, the sequences generated went through a filtering process to obtain qualified reads. Trimmomatic was implemented to clip the 3′ adaptor sequence, trim or remove the reads according to the quality score, and discard trimmed reads shorter than 18 nucleotides. Qualified reads remaining after filtering out the low-quality data were analyzed using miRDeep2 for aligning reads to the reference genome. Only reads that mapped perfectly to the genome five or less times were used for miRNA detection, since miRNAs usually map to few genomic locations. MiRDeep2 estimates expression levels of known miRNAs (which reads mapping to miRBase), and identifies novel miRNAs. Differentially expressed miRNAs that met a >2-fold change and *p*-value ≤ 0.05 cut-off were identified among miRNA profiles. Furthermore, miRNA-targeted genes were identified by using the online resource miRDB. miRNA-targeted genes that significantly participate in biological responses and regulate signaling pathways were investigated via over-representation analysis. RNAhybrid software (RNAhybrid—Free Software Directory (https://www.fsf.org/))was used to calculate the free energy of the miRNA-mRNA duplex and the predicted miRNA–mRNA hybrid structure.

### 2.6. Statistical Analysis

The data were recorded as mean ± SD. Statistical significance was determined using a one-way analysis of variance (ANOVA) using the SAS general linear model procedure (SAS Inc., Cary, NC, USA), followed by ANOVA using Duncan’s test. The results were considered statistically significant at *p* < 0.05. Microbiota assay was carried out, according the methods of our recent study [9]. For β diversity of gut microbiota analysis, different distance matrices were evaluated using principal coordinate analyses (PCA) assay. The value corresponding to the heatmap represents the z-score obtained by the abundance of each species in all groups. The z-score of a sample in a certain classification is the value of the average abundance of the sample in the category and all samples in the classification. A heatmap and a heat tree were used to illustrate the diversity change in the bacteria.

## 3. Results

### 3.1. The Regulation of L. reuteri-Derived EVs on Microbiota in an In Vitro Cultured Intestinal Bacteria System

The cell–cell, cell–microbe, and microbe–microbe communication primarily occur through the exchange of informative molecules carried by EVs secreted by cells or microbes. These information molecules include proteins, small RNA, microRNA, DNA, etc., facilitating interactions to achieve communication [16]. There is already evidence indicating that the EVs secreted by microbes interactively influence the gut microbiota [17]. Gut microbiota and intestinal function have been regulated by LAB through EVs secretion. However, the impact of these EVs from LAB on intestinal microbes remains unclear. There is a lack of detailed literature exploring the mechanisms involved in this interaction. Currently, fewer studies have conducted functional assessments on the anti-bacterial activity of LAB-derived EVs [18,19]. Our recent study showed that *L. reuteri*-derived EVs can regulate the intestinal flora of *O. fasciatus* and reduce intestinal pathogenic bacteria (*Acinetobacter baumannii*); we further determined that the inhibitory ability of *L. reuteri*-derived EVs against pathogenic bacteria occurs mainly through *L. reuteri*-derived EVs, containing small RNA/microRNA [9]. In recent years, research has indicated that the intestinal epithelial cells of hosts regulate the growth and survival of target bacteria. The primary mechanism involves specific microRNAs contained in EVs secreted by intestinal epithelial cells, which interact with genes corresponding to the microbes. This interaction allows specific microbes to proliferate in the intestine; for example, intestinal cells produce miR-515-5p and miR-1226-5p, which respectively regulated the gene expression and growth in *Fusobacterium nucleatum* and *Escherichia coli* [20]. These results not only demonstrate the crucial role of the physiological state of intestinal cells in shaping the composition of the intestinal microbiota, but also suggest that when investigating how *L. reuteri*-derived EVs regulate the gut microbiota, a host-free microbial culture system is necessary to avoid interference from host factors. Recently, a similar concept has been used, involving evaluating the improvement of the intestinal microbiota in shrimp (*Litopenaeus vannamei*) through an in vitro cultured intestinal bacteria system when exposed to probiotics [21].

**Figure 1:**
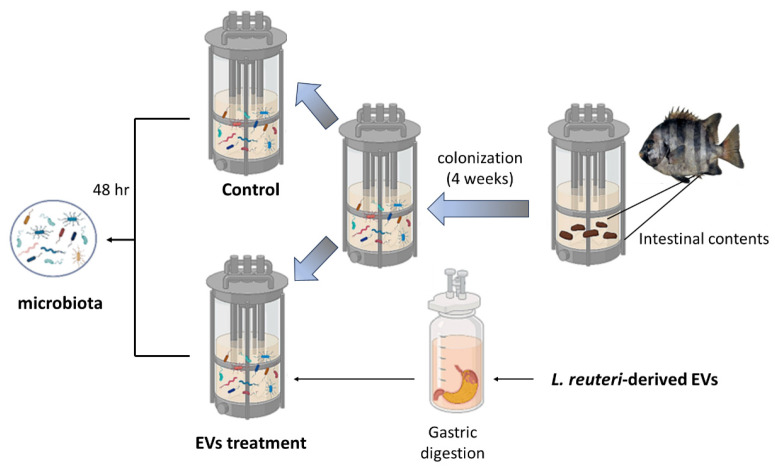
Overview of the in vitro cultured intestinal bacteria system used in the study. The feces from intestinal contents were collected and regularly cultured in the fermenter (in vitro cultured intestinal bacteria system). After colonization, *L. reuteri*-derived EVs were pre-treated with gastric juice for 4 h, and condition medium was collected and transferred to an intestinal microbiota simulator, and microbiota sequencing was performed after co-cultivation for 48 h. EV: *L. reuteri*-derived EVs.

We have used an in vitro cultured intestinal bacteria system to create a host-free model to evaluate the potential of *L. reuteri*-derived EVs for the regulation of intestinal microbiota, as shown in Figure 1. Firstly, the intestinal contents of *O. fasciatus* was collected and homogenized, and this fecal solution was inoculated into the fresh culture broth and incubated at 30 °C to adaptation for 4 weeks under anaerobic conditions. In addition, the *L. reuteri*-derived EVs were added into an in vitro cultured intestinal bacteria system for 48 h, and the microbial community was sequenced.

**Figure 2:**
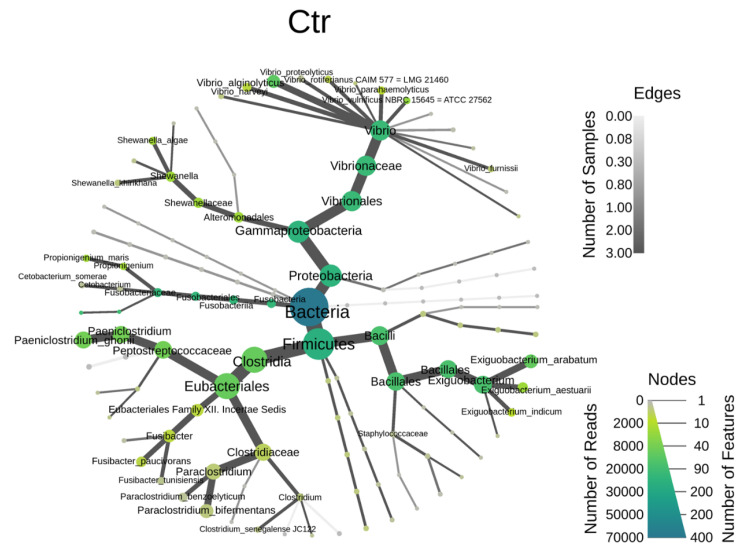

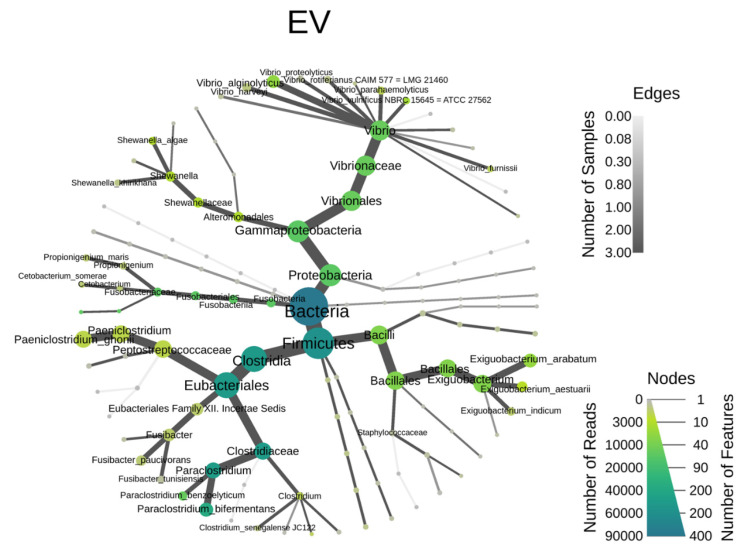
The heat trees obtained from analysis of bacteria microbiota in control and *L. reuteri*-derived EVs groups (*n* = 3). Ctr: control; EV: *L. reuteri*-derived EVs.

The heat tree was used to illustrate the diversity change in the bacteria, as shown in Figure 2. Our results implied that the identification of the bacterial taxonomy was similar between the control and the *L. reuteri*-derived EVs treatment groups, suggesting that different types of microorganisms within the system exhibit stability; the majority of intestinal bacteria can thrive and remain stable in the in vitro cultured intestinal bacteria system.

**Figure 3:**
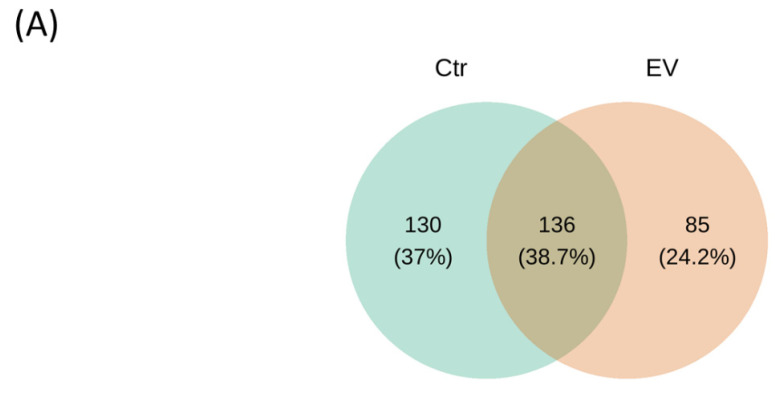

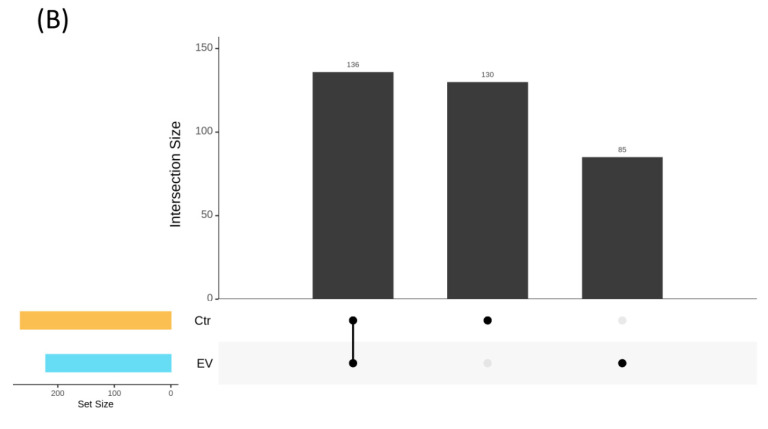
UpSetPlot illustrating the unique and shared bacterial taxa between control and EV-treated groups. (**A**) Venn diagrams of analysis for microbiota OTU in the in vitro cultured intestinal bacteria system. (**B**) Statistical analysis of microbial similarity and differences (n = 3). Ctr: control; EV: *L. reuteri*-derived EVs.

Figure 3A shows the Venn diagrams after cluster analysis in the control and EVs treatment groups, and Figure 3B shows the UpSetPlot results in the control and EVs treatment groups. There are 136 different types of bacteria (amplicon sequence variants, ASVs), both in the control and *L. reuteri*-derived EVs treatment groups in the in vitro cultured intestinal bacteria system. However, the 130 and 85 ASV numbers were only found in the control and *L. reuteri*-derived EVs treatment groups, respectively.

**Figure 4:**
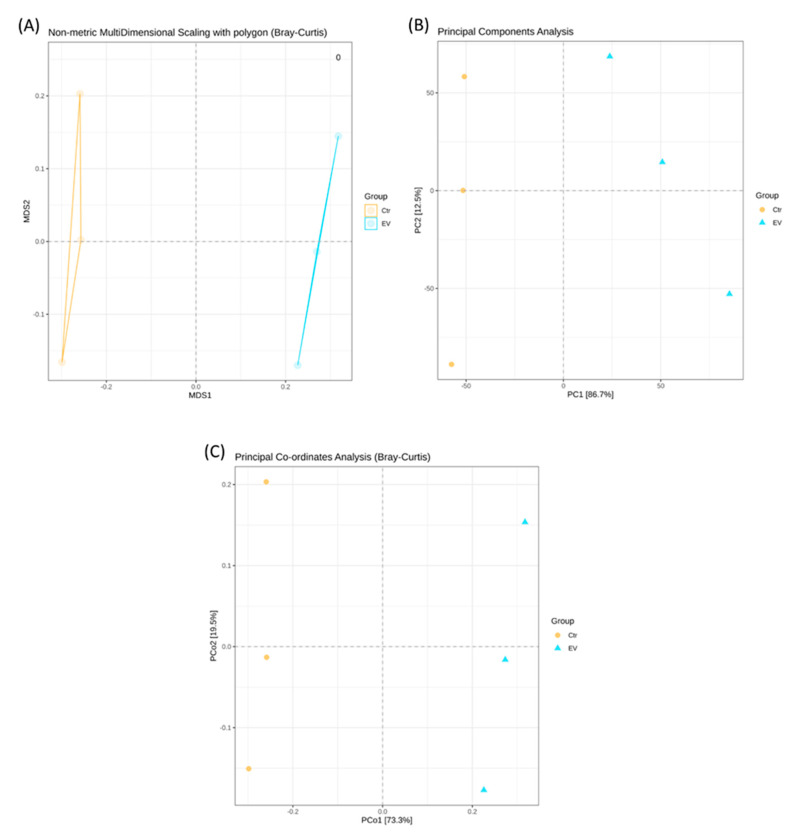
The microbial beta diversity obtained by (**A**) non-metric multidimensional scaling (NMDS), (**B**) principal components analysis (PCA), and (**C**) principal co-ordinates analysis (PCoA) in the in vitro cultured intestinal bacteria system (n = 3). Ctr: control; EV: *L. reuteri*-derived EVs.

The beta diversity of the microbiota obtained from the in vitro cultured intestinal bacteria system, with or without *L. reuteri*-derived EVs treatment, was analyzed by non-metric multidimensional scaling (NMDS) (Bray–Curtis), principal components analysis (PCA), and principal co-ordinates analysis (PCoA) (Bray–Curtis), as shown in Figure 4. It was determined that the widest distribution occurred between the control and the *L. reuteri*-derived EVs treatment groups.

### 3.2. The Target Bacteria in an In Vitro Cultured Intestinal Bacteria System Using L. reuteri-Derived EVs Regulation 

**Figure 5:**
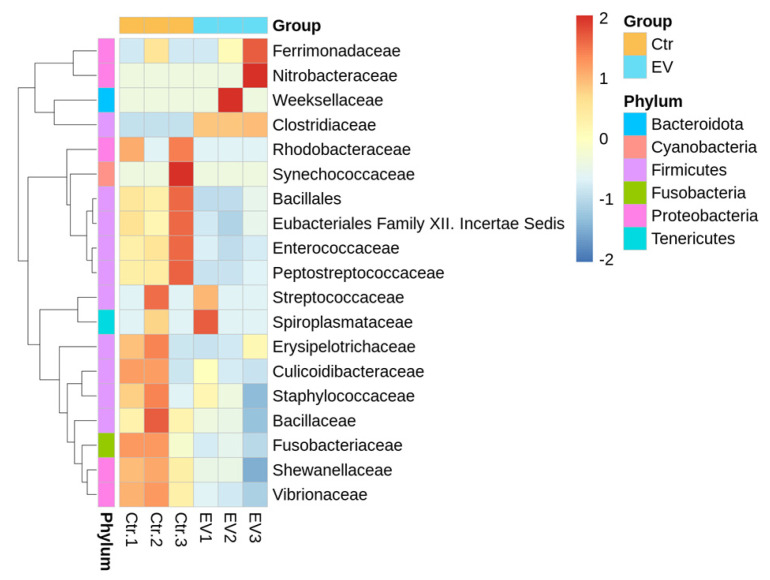
The heatmap obtained from analysis of microbiota (family level) in the in vitro cultured intestinal bacteria system (n = 3). Ctr: control; EV: *L. reuteri*-derived EVs.

From the perspective of the family for taxa analysis, the heatmap analysis for the bacterial community is shown in Figure 5. The results indicate most of the bacteria taxa were decreased by *L. reuteri*-derived EVs treatment, including *Rhodobacteraceae, Bacillales, Enterococcaceae, Peptostreptococcaceae, Erysipelotrichaceae, Culicoidibacteraceae, Staphylococcaceae, Bacillaceae, Fusobacteriaceae, Shewanellaceae,* and *Vibrionaceae*.

**Figure 6:**
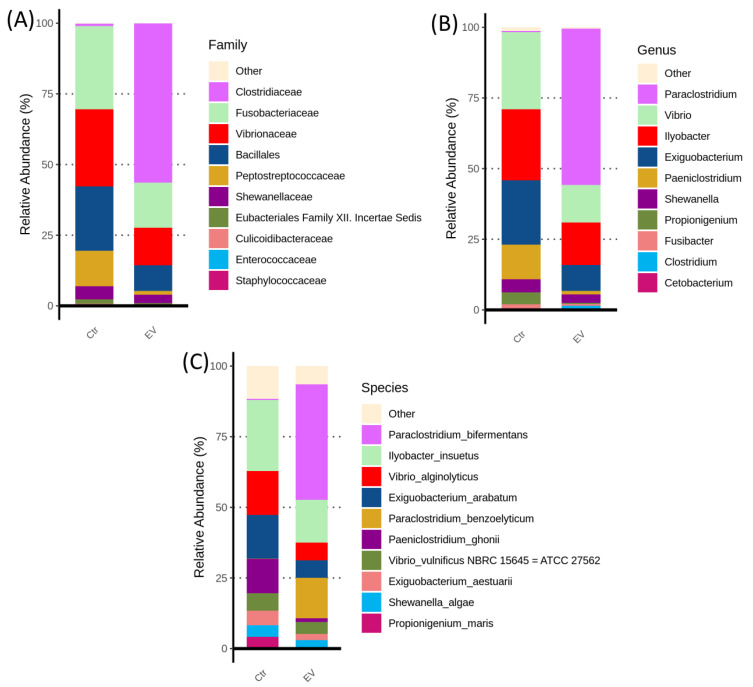
Top 10 classifications for (**A**) family, (**B**) genus, and (**C**) species levels of microbiota composition in the in vitro cultured intestinal bacteria system (n = 3). Ctr: control; EV: *L. reuteri*-derived EVs.

We analyzed the most abundant bacteria (top 10) in the in vitro cultured intestinal bacteria system, with or without *L. reuteri*-derived EVs treatment. The results (family) indicated that the levels of *Fusobacteriaceae, Vibrionaceae, Bacillales,* and *Peptostreptococcaceae* were reduced by *L. reuteri*-derived EVs treatment (Figure 6A). The populations of *Vibrio, Exiguobacterium,* and *Paeniclostridium* were markedly suppressed after treatment with *L. reuteri*-derived EVs (Figure 6B). Interestingly, *Vibrio alginolyticus, Exiguobacterium arabatum,* and *Paeniclostridium ghonii* are present in high proportions in the control group, but are inhibited by *L. reuteri*-derived EVs (Figure 6C).

**Figure 7:**
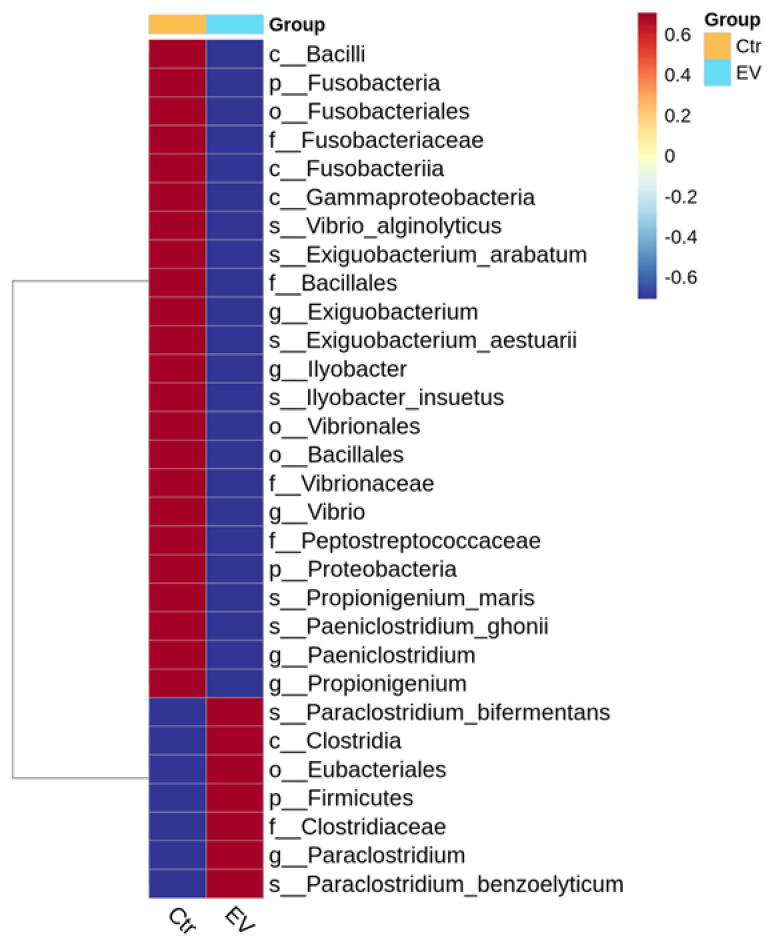
Biomarkers of microbiota composition in the in vitro cultured intestinal bacteria system according to linear discriminant analysis effect size (LEfSe) analysis (n = 3). Ctr: control; EV: *L. reuteri*-derived EVs.

We also used the statistical linear discriminant analysis effect size (LEfSe) method to determine the significance of differences in the species composition and community results of the grouped samples. A distribution histogram showing the biomarker taxa significantly affected by *L. reuteri*-derived EVs treatment is shown in Figure 7. Among the three bacteria, *Vibrio alginolyticus, Exiguobacterium arabatum,* and *Paeniclostridium ghonii*, are inhibited by *L. reuteri*-derived EVs in the in vitro cultured intestinal bacteria system, and only *Vibrio alginolyticus* is a common pathogen. The results indicated that *L. reuteri*-derived EVs may have regulatory potential against *Vibrio* spp.

**Figure 8:**
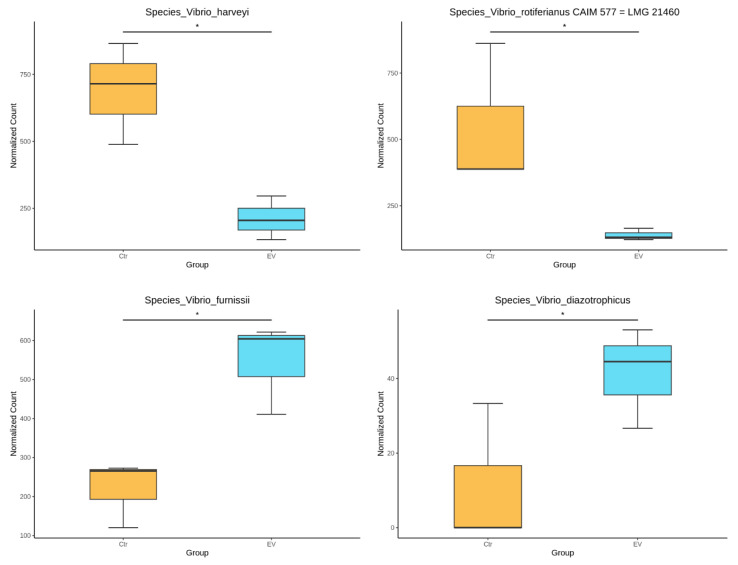
The analysis regarding other *Vibrio* species (*V. harveyi, V. rotiferianus, V. fumissii,* and *V. diazotrophicus*) found in the in vitro cultured intestinal bacteria system (n = 3). Ctr: control; EV: *L. reuteri*-derived EVs. The significant difference was shown by * (*p* < 0.05).

We further analyzed whether other *Vibrio* species found in the in vitro cultured intestinal bacteria system would also be affected by *L. reuteri*-derived EVs. Small amounts of *Vibrio harveyi*, *Vibrio rotiferianus*, *Vibrio furnissii*, and *Vibrio diazotrophicus* were detected in the in vitro cultured intestinal bacteria system. Our results revealed that *L. reuteri*-derived EVs could inhibit *V. harveyi* and *V. rotiferianus*, while increasing *V. furnissii* and *V. diazotrophicus* (Figure 8).

### 3.3. Prediction of Physiological and Growth-Related Genes of V. alginolyticus Affected by Small RNA Contained in L. reuteri-Derived EVs 

To investigate the mechanism by which *L. reuteri*-derived EVs inhibit *V. alginolyticus*, we further sequenced the small RNA content contained in *L. reuteri*-derived EVs. Subsequently, we used the *V. alginolyticus* gene database to predict the genes and their binding positions that are likely to be interfered with by the small RNAs contained in *L. reuteri*-derived EVs.

In *L. reuteri*-derived EVs, small RNAs, with approximately 250 read numbers each, can interfere with the expression of five genes in *V. alginolyticus*, including LysR, pirin, MIpA/OmpV, CatB, and aspartate-semlaldehyde dehydrogenase (Table 1). These small RNAs are capable of binding to different positions on the target genes, thereby interfering with their expression in *V. alginolyticus* (Tables S1–S5). We hypothesized that the potential of *L. reuteri*-derived EVs to inhibit *V. alginolyticus* may be related to their ability to negatively regulate the expression of these genes.

## 4. Discussion

Many probiotics have been applied to aquaculture organisms in recent years [18,22,23], not only to optimize the intestinal environment and regulate the intestinal microbiota, but also to reduce the quantity of pathogenic bacteria (especially *V. alginolyticus*) in *O. fasciatus* [24]. The animal intestine harbors various types of bacteria which can successfully adhere to the surface of intestinal mucosal cells and subsequently proliferate in the intestine. However, these bacteria engage in different interactions, including competitive exclusion, the barrier effect, bacterial interference, and antagonism, all of which significantly reduce the benefits of probiotics in aquaculture organisms [25]. 

The gut microbiota of terrestrial animals remains stable within their intestines. However, the environment strongly influences the gut microbiota of aquaculture organisms. This is because seawater flows through the digestive tracts of aquaculture organisms, and their intestines are relatively shorter compared to those of terrestrial animals. As a result, most microorganisms can only briefly remain in their intestines. Additionally, since aquaculture organisms are mostly poikilothermic (cold-blooded), changes in temperature also affect their gut microbiota [26]. Based on the above information, the benefits of administering probiotics to aquaculture organisms in the form of live bacteria may be diminished. Moreover, a study has challenged the conventional understanding of probiotics, revealing that oral probiotics might negatively impact the recovery of gut microbiota [27]. Traditionally, studies on gut microbiota have relied on fecal samples, but this research collected samples from different parts of the digestive tract and compared the microbiota and functional genes of these samples with those of the homologous fecal sample. The results showed that the microbiota or functional gene expression in all regions of the intestinal samples differed significantly from those in the fecal microbiota. Moreover, probiotics were continuously administered the study participants, and it was determined that in nearly half of the subjects, the ingested probiotics were directly excreted through feces. In the other half, a small number of probiotics were allowed to “temporarily” colonize the intestinal mucosa. The colonization by probiotics was temporary and ceased once the product consumption stopped. Subsequent experiments revealed that the ability of probiotics to colonize was highly selective; in other words, it was the host’s gut microbiota that determined whether the probiotics could remain in the intestine [27]. In aquaculture, antibiotics are often used for disease treatment, which can severely disrupt the gut microbiota. Research has shown that after using antibiotics to disrupt the gut microbiota of test subjects, administering live probiotics significantly increased the colonization of probiotics. In some cases, the probiotics remained effective for over five months. However, the study also found that in subjects where probiotics were able to colonize extensively, the speed of microbiota reconstitution (recovery of gut diversity) was significantly reduced [28].

Existing literature reports that extracellular vesicles (EVs) secreted by Gram-positive bacteria can inhibit the growth of Gram-negative bacteria. Conversely, EVs secreted by Gram-negative bacteria also inhibit the growth of Gram-positive bacteria [29]. On the other hand, EVs isolated from Gram-positive bacteria can stimulate the growth of their own bacteria, while EVs from Gram-negative bacteria also have the ability to enhance the distribution of their own bacterial populations [30]. These research findings indicate that EVs from microorganisms regulate the microbial flora (microbiota) in natural environments or within organisms, either synergistically or antagonistically [31]. For example, EVs secreted by *Pseudomonas aeruginosa* carry enzymes capable of degrading peptidoglycan, and these EVs have been confirmed to be toxic to both Gram-positive and Gram-negative bacteria [29].

Research has found that EVs produced by microbiota carry various digestive enzymes and diverse compounds. These EVs can influence the host through several mechanisms, including immune regulation and signaling pathways. Additionally, EVs can alter the signaling molecules of the intestinal barriers, thereby affecting organ function [32]. Administering EVs secreted by specific bacterial strains can regulate immune signaling pathways, host nutrition, and the production of bacterial metabolites [33]. Many EVs secreted by bacteria have the ability to kill competitive microorganisms, thereby promoting their own growth. For example, EVs from *Pseudomonas aeruginosa* can exert antibacterial effects by carrying murein hydrolase, which degrades the peptidoglycan of both Gram-positive and Gram-negative bacteria [29]. EVs from different species may participate in interactions between host cells, between host cells and microorganisms, and between the microorganisms themselves, facilitating communication between different species [34], suggesting that microbial EVs have the ability to regulate microbiota. If applied in the biotechnology and aquaculture industries, they could potentially replace the use of antibiotics for disease prevention and control. Our recent findings have found that *L. reuteri* offers potential for accelerating growth rates, improving intestinal microbiota composition, and enhancing immune activity in *O*. *fasciatus*; we also found that EVs derived from *L. reuteri* can reduce the quantity of intestinal pathogenic bacteria in *O. fasciatus* [9].

EVs exhibit the potential for encapsulating and transporting different cargo, including proteins, peptides, enzymes, RNA, and DNA. Various organisms (i.e., microorganisms, plants, and animals) secrete EVs, thereby interacting with each other through the exchange of EVs-contained cargo to regulate target gene expression [15,35]. In the past, we have reported that LAB-derived EVs possess the ability to inhibit the growth of pathogenic bacteria [9,18,19]. Moreover, the application of EVs has been widely discussed in regards to the aquaculture industry [36] and antibiotics resistance [37]. Marine algal cell-derived EVs showed biological protection effects [38], in which the small RNA cargo of *Emiliania huxleyi*-derived EVs modulate host–virus dynamics, suggesting the importance of EVs for the microbial interaction in the marine environment [39].

This study utilized a fermenter to establish a simulated system of intestinal microbiota (in vitro cultured intestinal bacteria system) [21]. The advantage of this system lies in its ability to exclude the interference of the host on the intestinal microbiota. Therefore, this model enables a clear investigation of the regulatory effects of *L. reuteri*-derived EVs on a bacterial flora. The intestinal microbiota of *O. fasciatus* was cultured in an in vitro cultured intestinal bacteria system. After treatment with *L. reuteri*-derived EVs, significant changes were observed in the bacterial composition, specifically, a significant decrease in the quantity of *Vibrio* species.

We sequenced the small RNA in *L. reuteri*-derived EVs and evaluated the regulatory potential of these small RNAs on target genes of *V. alginolyticus* using a predictive strategy. The results showed that *L. reuteri*-derived EVs contain a significant amount of small RNAs that correspond to the genes of LysR, pirin, MIpA/OmpV, CatB, and aspartate-semialdehyde dehydrogenase in *V. alginolyticus*. LysR family activator-regulated major facilitator superfamily transporters are involved in *Vibrio cholerae* antimicrobial compound resistance and intestinal colonization [40]. The virulence and growth were limited in *V. cholerae* under low pH and anaerobiosis conditions, while the LysR expression was suppressed [41]. Pirin protein is considered to be associated with the survival of bacteria and prokaryotes, regulating cellular ATP production and growth to the stationary phase (high cell density) [42], as well as interacting with the pyruvate dehydrogenase E1 subunit and pyruvate catabolism [43]. The MIpA/OmpV protein plays an important role in the development of antibiotic resistance; when the expression of this gene is disrupted, the bacterial resistance to the environment is weakened, leading to reduced survival rates [44]. The reduction of the *V. alginolyticus* population in the environment is due to the attenuation of the MIpA/OmpV gene expression [45]. The *Cat*B gene has been found to enhance the tetracycline resistance capability of *Vibrio spp.* [46], and the reduction of CatB expression results in the suppression of *Vibrio vulnificus* growth [47]. The basic mechanism of aspartate-semialdehyde dehydrogenase in *V. cholerae* has been investigated [48]. The inhibition of aspartate-semialdehyde dehydrogenase affects amino acid metabolism and physiological function [49].

Probiotic EVs exhibit significant potential to replace or reduce the use of antibiotics, promote the growth rate of aquaculture species such as shrimp, and increase harvest yields and economic benefits. In addition to addressing food safety concerns, they can also reduce the overfishing of small fish used as feed and mitigate the environmental harm caused by antibiotics, contributing to a greener aquaculture industry. Utilizing fermentation liquid, which contains a large number of probiotic EVs, which are considered waste by food fermentation plants, as a raw material for development could unite aquaculture farmers, the biomedical industry, and food fermentation plants for applications in biological disease prevention.

## 5. Conclusions

In summary, these results indicate that *L. reuteri*-derived EVs possess inhibitory potential against *Vibrio*, mediated by small RNA interfering LysR, pirin, MIpA/OmpV, CatB, and aspartate-semialdehyde dehydrogenase. Future research should focus on the in vivo validation of these findings and exploring the therapeutic potential of *L. reuteri*-derived EVs in aquaculture

## Figures and Tables

**Table 1:** The quantity of *L. reuteri*-derived EVs containing small RNA can interfere with the target genes of *V. alginolyticus*.

Gene ID	Gene Name	Small RNA (Reads)
NAL94_RS00020	LysR	255
NAL94_RS04885	pirin	1700
NAL94_RS06120	MIpA/OmpV	348
NAL94_RS07195	CatB	571
NAL94_RS12345	aspartate-semlaldehyde dehydrogenase	2020

## Data Availability

The original contributions presented in the study are included in the article/Supplementary Materials, further inquiries can be directed to the corresponding author/s.

## Supplementary Materials

The following supporting information can be downloaded at: https://www.mdpi.com/article/10.3390/ani14121792/s1, Table S1: The prediction of small RNA interference with LysR; Table S2: The prediction of small RNA interference with pirin; Table S3: The prediction of small RNA interference with OMP; Table S4: The prediction of small RNA interference with CatB; Table S5: The prediction of small RNA interference with aspartate-semialdehyde dehydrogenase.
